# Phenotypes Associated With MEN1 Syndrome: A Focus on Genotype-Phenotype Correlations

**DOI:** 10.3389/fendo.2020.591501

**Published:** 2020-11-18

**Authors:** Chiara Mele, Monica Mencarelli, Marina Caputo, Stefania Mai, Loredana Pagano, Gianluca Aimaretti, Massimo Scacchi, Alberto Falchetti, Paolo Marzullo

**Affiliations:** ^1^ Department of Translational Medicine, University of Piemonte Orientale, Novara, Italy; ^2^ Istituto Auxologico Italiano, IRCCS, Division of General Medicine, S. Giuseppe Hospital, Piancavallo, Italy; ^3^ Istituto Auxologico Italiano, IRCCS, Laboratory of Molecular Biology, S. Giuseppe Hospital, Piancavallo, Italy; ^4^ Department of Health Sciences, University of Piemonte Orientale, Novara, Italy; ^5^ Division of Endocrinology, University Hospital “Maggiore della Carità”, Novara, Italy; ^6^ Istituto Auxologico Italiano, IRCCS, Laboratory of Metabolic Research, S. Giuseppe Hospital, Piancavallo, Italy; ^7^ Division of Endocrinology, Diabetology and Metabolism, Department of Medical Sciences, University of Turin, Turin, Italy; ^8^ Istituto Auxologico Italiano, IRCCS, Rehabilitation Unit, S. Giuseppe Hospital, Unit for Bone Metabolism Diseases, Verbania, Italy; ^9^ Diabetes & Lab of Endocrine and Metabolic Research, Dept. of Clinical Sciences & Community Health, University of Milan, Milan, Italy

**Keywords:** MEN1, genotype, phenotype, mutations, tumors

## Abstract

Multiple endocrine neoplasia type 1 (MEN1) is a rare autosomal dominant inherited tumor syndrome, associated with parathyroid, pituitary, and gastro-entero-pancreatic (GEP) neuroendocrine tumors (NETs). MEN1 is usually consequent to different germline and somatic mutations of the *MEN1* tumor suppressor gene, although phenocopies have also been reported. This review analyzed main biomedical databases searching for reports on *MEN1* gene mutations and focused on aggressive and aberrant clinical manifestations to investigate the potential genotype-phenotype correlation. Despite efforts made by several groups, this link remains elusive to date and evidence that aggressive or aberrant clinical phenotypes may be related to specific mutations has been provided by case reports and small groups of MEN1 patients or families. In such context, a higher risk of aggressive tumor phenotypes has been described in relation to frameshift and non-sense mutations, and predominantly associated with aggressive GEP NETs, particularly pancreatic NETs. In our experience a novel heterozygous missense mutation at *c.836C>A* in exon 6 was noticed in a MEN1 patient operated for macro-prolactinoma, who progressively developed recurrent parathyroid adenomas, expanding gastrinomas and, long after the first MEN1 manifestation, a neuroendocrine uterine carcinoma. In conclusion, proof of genotype-phenotype correlation is limited but current evidence hints at the need for long-term interdisciplinary surveillance in patients with aggressive phenotypes and genetically confirmed MEN1.

## Introduction

Multiple endocrine neoplasia type 1 (MEN1) syndrome (OMIM#131100) is a rare autosomal dominant inherited tumor syndrome with high penetrance, that is typically associated with parathyroid, pituitary, and gastro-entero-pancreatic (GEP) neuroendocrine tumors (NETs), either functioning or nonfunctioning (1, 2). Adding to the intrinsic burden of MEN1, a different combination of endocrine and non-endocrine tumors may develop, i.e. carcinoids (thymic, bronchial), adrenocortical tumors, facial angiofibromas, lipomas, and collagenomas (3–5).

The clinical behavior of MEN1 largely depends on tumor spread and histological features, as well as type and degree of hormone hypersecretion, risk of tumor recurrence, and duration of surveillance (6). The *MEN1* gene (NM_130799.2) is a tumor-suppressor gene located on chromosome 11q13 (7, 8). This gene spans approximatively 9,000 bp of genomic DNA containing 10 exons and is transcribed into a 2.8 kb mRNA with the translational start codon (ATG) in exon-2 and the stop-codon in exon-10. The protein product of the *MEN1* gene is called MENIN, of which different isoforms have been reported: long isoform 1, 615 amino acids, chosen as the canonical sequence, short isoform 2, 610 amino acids length, and isoform 3 consisting of 575 amino acids (9). MENIN nuclear localization sequences (NLSs) are located in its C-terminal region and directly interact with DNA in a sequence-independent manner to serve as a scaffold protein that controls gene expression and cell signaling (10). The majority of patients with the inherited form of the disease carry germline mutations of the *MEN1* gene (11). Germline heterozygous inactivating mutations were found in the coding region of the *MEN1* gene in index cases and affected family members, together with loss of heterozygosity (LOH) for markers at the *MEN1* gene locus in their tumors as expected for a causative tumor suppressor gene (12–14). To date, many germline or somatic mutations have been described both in MEN1 families and sporadic cases (15–18).

*MEN1* gene mutations are distributed throughout the coding region without particular hot spot regions. Since the original description of the *MEN1* gene, different germline MEN1 mutations have been identified across its coding sequences (6, 15). Approximatively 10% of the *MEN1* mutations arise *de novo* and are later transmitted to following generations (19). More than 10% of mutations are nonsense mutations, 40% frameshift insertions and deletions, 25% missense mutations while 11% are splice site defects (20). Generally, the MEN1-related tumors occurrence requires inheritance of a germline mutation of *MEN1* gene together with a somatic mutation in the DNA of tumor, leading to LOH, according to the two hits model by Knudsons’ (13, 15). However, in multifocal MEN1-gastrinomas, with Zollinger–Ellison syndrome, it has been described that LOH at 11q13 region can be found in less than 50% of patients, and within the same patient different tumoral foci frequently exhibited different patterns of LOH, ranging from LOH limited only to 11q13 to loss of the whole chromosome or no LOH, suggesting that each focus of gastrinoma may arise by an independent second hit (21). It has been also hypothesized that preneoplastic G-cells hyperplastic lesions, and similarly to what described in somatostatin‐secreting tumors from the same patients, may retain both MEN1 alleles and that LOH and/or *MEN1* gene mutation, reported to be present in 54% of tumors lacking evidence of LOH, may account for the initiation step of neoplastic lesions (21). Lack of mutations have been reported in 5–25% of patients with a clinical diagnosis of MEN1, constituting the so-called “phenocopies” (15, 22, 23). Of note, a recent study on 189 patients with typical MEN1 phenotype described a higher prevalence (74%) of mutation-negative cases than previously reported (24).

Interest has increasingly focused on the genetic mutations associated with MEN1 hinting at potential genotype-phenotype correlations. To date, genotype-phenotype correlations in MEN1 have been difficult to assess, and the nature of mutations appears to play a null role in clinical MEN1 features, i.e. age of onset, multicentricity, recurrence or markers of aggressiveness (25, 26). Opposed to MEN1 syndrome, the existence of a strong genotype-phenotype correlation has been demonstrated in genetic studies of MEN2 syndrome (1). In the present narrative review, we sought to summarize evidences from the literature on peculiar *MEN1* gene mutations with a focus on aggressive and/or aberrant clinical presentations. The search for relevant original publications and case reports written in English was performed on PubMed, Embase, Scopus, and Google Scholar using the NCI Dictionary of Cancer Terms: multiple endocrine neoplasia type 1 OR MEN1 AND phenotype OR genotype, multiple endocrine neoplasia type 1 OR MEN1 AND genotype-phenotype correlation, multiple endocrine neoplasia type 1 OR MEN1 AND aggressive, multiple endocrine neoplasia type 1 OR MEN1 AND aberrant. As an index case, we describe a MEN1 patient harboring a novel sporadic germline mutation and late aggressive clinical features, so as to further discuss the potential clues underlying a genotype–phenotype correlation in MEN1.

## Gene Mutations Involved in the Pathogenesis of MEN1

Since the *MEN1* gene has been identified, 1,698 mutations have been described (17). Of these, approximately 85% are germline and 15% are somatic mutations (15, 27). Germline *MEN1* mutations consist of about 460 different mutations, distributed throughout the 1,830 bp coding region and splice sites of the *MEN1* gene (27).

Different types of *MEN1* gene mutations and their frequencies have been summarized in **Table 1**. These mutations are dispersed throughout the coding region of the gene rather than being clustered (27). Approximately 75% of *MEN1* mutations are inactivating, as it often happens for tumor suppressor genes (17). A few mutations repeatedly described in unrelated families, refer to 9 sites in the *MEN1* gene, and account for over 20% of all germline mutations (27). These mutations are recurrent and possibly represent hot spots. Deletion or insertion of these hot spots elements has also been reported in association with DNA sequence repeats, DNA stretches of long strips of either single nucleotides or shorter repeat elements (28).

**Table 1:** Different types of gene mutations associated with MEN1 and their frequencies.

Type of MEN1 mutations	Frequency
**Large rearrangements**	**6.2%**
Deletions	6.0%
Duplications	0.2%
**Small rearrangements**	**90.8%**
Deletions	21.3%
Insertion	14.7%
Splite sites	10.8%
Point mutations	42.2%
Nonsense	17.0%
Missense	25.1%
**Mid-intronic variations**	**3.0%**

Data are gathered from the UMD-MEN1 mutations database (17).

Approximatively 5–10% of MEN1 patients may show no evident germline mutations in the *MEN1* gene coding region (12, 14, 28–31) but they may harbor large mutations or deletions in the promoter or untranslated regions, which have not been described to date (27). Large deletions are hard to detect by conventional Sanger sequencing, and multiplex ligation-dependent probe amplification (MLPA) (32) or next-generation sequencing (NGS) technology, specifically whole genome testing, or long-read sequencing, the latter enabling also detection of intronic or promoter mutations, are predicted to more accurately extrapolate information on such large gene rearrangements (33).

Additional genetic mechanisms have been recently investigated for their possible involvement in MEN1 phenotype (27). Particular interest has focused on the p27Kip1 protein, which is encoded by the *CDKN1B* gene and is located downstream of MEN1-driven tumorigenesis (34). The V109G variant of p27 has been reported to influence the clinical manifestation of MEN1 subjects who carry truncating *MEN1* gene mutations (34). Carriers of both genetic variants, i.e. truncating *MEN1* mutation and *V109G* variant, show a more aggressive clinical behavior with a worse prognosis of the syndrome (35). These mechanisms will subsequently be detailed.

## The Classical Clinical Spectrum of MEN1

Clinical manifestations of MEN1 are predominantly associated with classical endocrine tumors and their relative secretion products (28). Typically, MEN1 is characterized by the presence of several endocrine tumors in the parathyroids, the pituitary gland and the GEP tract. Possible concomitant bronchial, thymic, type II gastric entero-chromaffin-Like (ECL) NETs, and adrenocortical tumors have also been reported. Likewise, a variable number of other endocrine and non-endocrine tumors have been described in the context of MEN1 phenotype, such as central nervous system (CNS) and cutaneous tumors, and will be subsequently summarized (36). MEN1 can affect all age groups, from 5 to 82 years (37, 38), although clinical and/or biochemical manifestations onset in nearly 95% of patients by the fifth decade (39). MEN1 affects both genders equally, and a recent series of 734 cases described a 57.8% female predominance (40).

### Parathyroid Tumors

Parathyroid tumors, resulting in primary hyperparathyroidism (PHPT), affect up to 95% of MEN1 patients and represent the first manifestation of the syndrome, with more than 85% of cases between ages 20 and 25 years (1, 39, 41, 42). PHPT manifests with hypercalcemia in 100% of affected patients by age 50 years (43). Compared to sporadic PHPT, bone disease and urolithiasis in MEN1-related PHPT reportedly show an early onset and higher severity (44–46). Interestingly, Kanazawa and colleagues demonstrated, in isolated Men1 knock-out osteoblasts model, that menin may play a key role in bone development, remodeling, and maintenance of bone mass *in vivo* (47), although in patients with germline *MEN1* mutations a specific genotype-phenotype correlation has never been described in this regard.

### GEP-NETs Tumors

GEP tumors occur in 70–80% of patients and mainly consist of gastrinomas (the most frequent functioning pancreatic tumor), glucagonomas, insulinomas, vaso-active intestinal peptidomas (VIPomas), and non-functioning tumors (28, 39, 42).

More than 80% of MEN1-associated gastrinomas exhibit, at pathology, multiple microgastrinomas within the first and second duodenum portion (48). Gastrinomas are usually associated with hypergastrinemia, increased gastric acid secretion and peptic ulcers, a clinical combination usually referred to as the Zollinger-Ellison syndrome (ZES) (22). Approximatively 50% of MEN1-associated duodenal microgastrinomas harbor LOH at the *MEN1* locus (49). MEN1-associated gastrinomas exhibit a malignant course and metastasize to local lymph nodes and liver in about 50% of cases, even before diagnosis (48, 50). Liver metastases negatively affect prognosis and survival, whereas lymph node metastases do not seem to affect the prognosis (51).

Interestingly, considering that most patients with ZES are treated for long periods with proton pump inhibitors (PPI), a debate has recently emerged on the potential sensitivity to PPI-related secondary hypergastrinemia, leading to a potential increased risk of gastric neuroendocrine tumors, as well as other tumors (52).

In the context of MEN1, insulinomas are the second most prevalent functioning pancreatic tumor. They are characterized by hypoglycemia and the typical Whipple’s triad, which is the first clinical manifestation of MEN1 syndrome in about 10% of subjects (36, 53). MEN1 insulinomas usually manifest as single benign lesions (54), often sized >1 cm, in the setting of multiple islet macroadenomas (55).

Glucagonomas and VIPomas are infrequent and often present as large lesions (>3 cm) with predominant benign behavior. Glucagonomas occur in fewer than 3% of subjects with MEN1 (56). Symptoms are often vague and the typical signs of skin rash, anemia, weight loss, and stomatitis can be absent, while mass effects can be present (22). In asymptomatic MEN1 patients, the presence of the tumor can be suspected upon pancreatic imaging in the presence of glucose intolerance and hyperglucagonemia (22). VIPomas are clinically characterized by watery diarrhea with achlorhydria and hypokalemia (57).

Non-functioning GEP NETs are the most frequent tumor types. They occur in approximately 55% of MEN1 subjects and are often multiple, asymptomatic or cause compressive symptoms (58). An accurate identification of non-functioning GEP NETs is clinically important for three main reasons: 1) these tumors could have a malignant course, which represent the most frequent cause of death in MEN1 patients; 2) several studies demonstrated that non-functioning GEP NETs are associated with a worse prognosis compared to other functioning tumors; 3) the absence of a clinical syndrome and specific biomarkers can result in delayed diagnosis, which increase the mortality rate (22, 59).

### Anterior Pituitary Tumors

Anterior pituitary tumors occur in about 30% of subjects and represent the first phenotypic manifestation of MEN1 in up to 42% of sporadic cases (28, 41, 42, 60). In 65–85% of MEN1 patients, pituitary tumors are represented by macroadenomas, a proportion that exceeds that recorded in sporadic tumors (55, 60). In about 30% of cases, pituitary tumors are locally invasive (60). Prolactinomas are the most prevalent pituitary adenomas in the context of MEN1 (65%), followed by somatotropinomas, ACTH-secreting tumors, and non-functioning tumors, the frequency of which is often overlooked (61). Clinical manifestations of pituitary adenomas in patients with MEN1 parallel those of sporadic tumors, hence they depend on hormone hypersecretion, tumor size, pathological features, and pituitary reserve. Depending on specific pituitary hormone hypersecretion, patients could manifest symptoms of hyperprolactinemia (e.g. galactorrhea, amenorrhea, and infertility in women, impotence and infertility in men) or develop somatic and metabolic alterations associated with Cushing’s disease or acromegaly. Large and/or invasive pituitary tumors could compress adjacent structures including the normal pituitary tissue and the optic chiasm, leading to hypopituitarism and/or visual disturbance (22).

### Other Endocrine Tumor Types

Adrenal cortical tumors are not rare in patients with MEN1, occurring in approximately one-fourth of patients with genetically confirmed MEN1 (5). Less than 10% of subjects with adrenal tumors show hormonal hypersecretion (62–64), mainly consisting of primary hyperaldosteronism and/or hypercortisolism (65). In 2002, Langer et al. conducted a clinical study with the aim of monitoring 66 patients with confirmed *MEN1* germline mutations in a screening program that included evaluation of the adrenal glands. They observed that patients with mutations in exons 2 and 10 of the *MEN1* gene develop adrenal lesions more often than subjects with other mutations of the *MEN1* gene. The malignant potential of MEN-1-related adrenal neoplasia is of clinical relevance (5).

Approximately 10% of MEN1 patients can develop thymic, bronchial, or type II gastric ECL carcinoids. Thymic NETs are aggressive malignant tumors that preferentially occur in male smokers (66). Their detection is largely dependent on imaging studies. In women, carcinoids are primarily multicentric and metasynchronous bronchial NETs and their course is generally indolent (66, 67). However, Lecomte et al. described cases of poorly differentiated and aggressive bronchial NETs, which are associated with an increased mortality (68).

### Non-endocrine Tumors

MEN1 patients can also develop lipomas, collagenomas, facial angiofibromas, CNS tumors including meningiomas and ependymomas, and smooth-muscle tumors, including leiomyomas (1, 4, 30, 39, 41, 51).

Skin tumors tend to be multiple and their diagnosis often precedes the onset of clinical hormone-dependent manifestations, thus contributing to early diagnosis of MEN1 (69, 70). Subcutaneous, pleural, visceral, or retroperitoneal lipomas (34%), facial angiofibromas (88%), and collagenomas (72%) may occur frequently in patients with MEN1 (1, 71). CNS tumors include asymptomatic meningiomas in 8% of MEN1 patients (70), while ependymomas and schwannomas affect about 1% of cases (72). Recently, the case of a MEN1-related mature teratoma and yolk sac testis tumor has been described (73).

## Aggressive and Aberrant Phenotype of MEN1

Cases of MEN1 phenotypes featured by aggressive or aberrant presentation, malignant evolution, and unfavorable clinical course have been reported in the literature and will be followingly summarized.

### Parathyroid Carcinoma

The presence of parathyroid carcinoma in association with MEN1 has been so far described in 16 cases (74–84). The clinical characteristics of these cases are reported in **Table 2**. In 2016, a single tertiary care center study conducted from 1997 to 2013 in a cohort of 348 patients with MEN1 syndrome, reported only one case of parathyroid carcinoma with a prevalence of 0.28% (80). In the same year, Christakis et al. collected 291 cases with a genetic and/or clinical diagnosis of MEN1 from the MD Anderson patients’ database (81). Hyperparathyroidism was diagnosed in 242 of these patients (83.2%) with two of them receiving a histopathologic diagnosis of parathyroid carcinoma which accounted for an overall prevalence of 0.8%, while 1 patient (0.4%) received a diagnosis of atypical parathyroid neoplasm. Then, progression to malignancy does not seem to be a prerogative of MEN1 parathyroid tumors. Interestingly, *MEN1* gene an inactivating mutation and a splicing mutation, previously identified in subjects who developed malignant lesions, has been reported, thus suggesting a possible genotype−phenotype association (89, 90).

**Table 2:** Summary of clinical features of 16 patients with parathyroid carcinoma and MEN1.

References	N° of cases	Age (years)	Gender	MEN1 phenotype
Wu et al. (85)	1	48	M	PC
				PT
Sato et al. (86)	1	51	F	PC
				PA
Dionisi et al. (87)	1	35	M	PC with mediastinal metastases
				Multiple PA
				pNET (gastrinomas)
				LI
Agha et al. (74)	2	69	F	PC with mediastinal metastases
				Lactotroph PT
				Non-functioning pNET
		32	M	PC
				pNET (gastrinomas and insulinoma)
Shih et al. (75)	1	53	F	Bilateral PC
				Bilateral PA
				PT
				pNETs (gastrinomas)
Kalavalapalli et al. (76)	1	40	F	PC with lung metastases
				PT with silent acromegaly
				Non-functioning pNET
Juodelé et al. (77)	1	39	F	Two PC
				pNETs (insulinomas)
				PT (prolactinoma)
				AA
				Multiple LI
del Pozo et al. (78)	1	50	M	PC
				pNETs (gastrinomas)
				AA
Lee et al. (88)	1	59	F	PC
				Two non-functioning PT
				AA
Singh Ospina et al. (80)	1	62	M	PC infiltrating the esophagus
				pNETs (gastrinomas)
				AA
				Multiple BC
Christakis et al. (81)	2	54	M	PC
				pNET
				BC
		55	M	PC
				PH
				pNET
				PT
				AA
Cinque et al. (82)	1	48	F	PC
				PA
				PH
				pNET
Omi et al. (83)	1	40	M	PC
				PH
				Non-functioning PT
				Non-functioning pNET
Song et al. (84)	1	49	M	PC
				pNET
				AA
				PT

PC, parathyroid carcinoma; PA, parathyroid adenoma; PH, parathyroid hyperplasia; PT, pituitary tumor; AA, adrenal adenoma; pNET, pancreatic neuroendocrine tumor; LI, lipoma; BC, bronchial carcinoid; TC, thymic carcinoid

### Malignant Insulinoma and Glucagonoma

Malignant insulinomas are rare, hence scant data exist on their prevalence and clinical presentation in MEN1 patients. In 2011, Hasani-Ranjbar et al. described a large family encompassing several members from three generations who were evaluated for MEN1. Genetic analysis was performed in all family members using PCR amplification of coding regions followed by direct sequencing. In three brothers presenting with hypoglycemia, the presence of insulinomas was confirmed and in two cases it was malignant, according to the surgery and pathology report. Two of these presented with hyperparathyroidism as well. Mutation screening revealed the presence of a two nucleotides deletion in the exon 2 resulting in a non-functional gene product (91). Recently, Novruzov et al. reported another case of malignant insulinoma with multiple metastatic lesions in the right lung, liver, and pancreas, in a 54-year-old man, who had a previous history parathyroid surgery and left thyroid lobectomy (92).

Only one case of malignant glucagonoma with cervical metastases has been reported to date in MEN1 (88).

### Pituitary Carcinoma

Only four cases of pituitary carcinoma have been described in association with MEN1 (79–82). In 2005, Benito and co-workers described the first case of a woman with a MEN1 associated gonadotroph carcinoma, who developed a temporal lobe metastasis (93). One year later, Gordon et al. presented the case of a 47-year-old male patient with MEN1 who was affected by parathyroid adenomas, non-functioning pancreatic tumors and a metastatic prolactinoma presenting as a cervical spinal cord lesion (94). Another case of malignant prolactinoma was described by Philippon and co-workers (95). Finally, Scheithauer et al. described the case of a 19-year-old man with a peculiar MEN1 phenotype, characterized by a parathyroid adenoma, pancreatic islet cell tumors in association with two enlarged hepatic hilar lymph nodes that were not biopsied, and a non-functioning pituitary mass with supra- and parasellar invasion, harboring craniospinal and systemic metastases (96).

### Adrenocortical Carcinoma

The incidence of adrenocortical carcinoma in patients with MEN1 has been reported as ranging between 1.4 and 6% (65, 97). The prevalence of adrenocortical carcinoma is reportedly 10 times higher in patients with adrenal tumors and MEN1 as compared to those with adrenal incidentalomas without MEN1 (64). Adrenocortical carcinoma can exhibit familial aggregation in MEN1 patients. In reviewing literature, we could document 22 cases of adrenocortical carcinoma associated with MEN1 (5, 63, 65, 97–105). The most peculiar and aggressive MEN1 phenotypes associated with adrenocortical carcinoma were recently described (104, 105). Wang et al. described the case of a 51-year-old man with MEN1-associated bilateral parathyroid adenoma, multiple pNETs, and left adrenocortical carcinoma, which metastasized to supraclavicular and mediastinal lymph nodes, bilateral lung, and uncinate process of pancreas (104). In the same year, Harada et al. reported the case of a 68-year-old woman with a complex MEN1 phenotype characterized by pancreatic insulinoma, breast cancer, non-functioning pituitary tumor, parathyroid adenoma, and a myxoid variant of adrenocortical carcinoma without metastases (105).

### Other Neoplasms

Ovarian NETs are rare and comprise 0.1% of all ovarian tumors. To date, few cases of primary ovarian NETs in women with MEN1 syndrome have been described in association with clinical manifestations of MEN1, but without genetic testing (106–109). More recently, clinical cases of ovarian NETs have been reported in genetically tested MEN1 cases. Jhawar et al. reported on a genetically confirmed case of MEN1 associated with an ovarian NET in a 33-year-old woman (109). Also, the case of an atypical ovarian carcinoid has been described as the first manifestation of an otherwise occult MEN1 syndrome in a 30-year old woman, who later developed a contralateral lesion two years after initial diagnosis (108). In this case, subsequent work-up allowed identification of simultaneous multifocal endocrine tumors involving parathyroids, thymus, adrenal glands, and pancreas, along with metastatic lesions in lymph nodes, liver, and bones.

### Non-endocrine Malignancies

Recent studies suggest a general role of menin in carcinogenesis that may affect the risk and clinical course of developing common non-endocrine neoplasms (22). The most frequent non-endocrine neoplasm in MEN1 is breast cancer, which is in the MEN1 setting characterized by earlier onset as compared to non-MEN1 patients (110). The calculated relative risk for breast cancer in MEN1 women is 2.83 (111), which advises to categorize the MEN1 gene as a moderate risk factor for breast cancer (112). Interestingly, it has been also described a patient harboring both *MEN1* and *BRCA1* germline mutations in whom the severity of the MEN1-related biochemical and clinical findings did not differ from those for other affected family members lacking the *BRCA1* mutation, but she did not develop any BRCA1-related malignancies (113). Other cancers that have been anecdotally described in association with MEN1 include hepatocellular carcinoma (114), melanoma (115–118), lung adenocarcinoma (103), renal cell carcinoma (119, 120), papillary thyroid cancer (120–122), and prostate cancer (120, 123).

## The Natural History of MEN1 Patients

MEN1 patients have an increased risk of premature death. Earlier studies reported an average life-span of 50 years in affected patients (124, 125). The leading cause of death is complications related to hypergastrinemia and hyperparathyroidism. Although the improvement of medical and surgical management has remarkably decreased the risk of premature deaths for such causes, survival curves in MEN1 patients remain significantly affected when compared to the general population (126). Main negative prognostic factors in MEN1 patients include clinical features, i.e. disease duration, presence of non-ZES functional syndromes, number of parathyroidectomies, occurrence of thymic carcinoid, family history and, in case of ZES, a previous acid-reducing surgery. Also, entity of hypergastrinemia and tumor features, such as pNETs size, liver metastases, distant metastases, number of lesions at imaging, and tumor growth, are also reported to play a role (126).

To date, there are only few prospective studies evaluating the long-term course and causes of death of MEN1 patients. In 2013, Ito et al. conducted a prospective study with the aim of describing the current course of MEN1 patients late in the disease history and the causes of death at present (126). Opposed to previous reports, this and other large MEN1 series reported that patients with MEN1 rarely die for causes related to hormone excess per se (126–129), while the likelihood of death increases in the presence of pNET tumors with a malignant behavior. Among these, gastrinomas account for more than one half of pNET-related deaths (130). Intriguingly, causes of death in one third of MEN1 patients do not involve MEN1-related causes, such as cardiovascular disease and neoplasms arising from other sites like colorectal, renal, lung, breast, and oral cancers (126–129). It remains unclear whether these are directly related to MEN1 and the role of menin in regulating growth-related processes has been hypothesized to play a role (4, 131, 132). Among the cardiovascular diseases, conditions at particular risk of complications include hyperparathyroidism and glucose intolerance/diabetes, these latter being reported to occur with a higher frequency in MEN1 patients (133–135).

## Diagnosis and Management of MEN1

Beyond work-up strategies for identification of MEN1-associated tumors, the simultaneous presence of at least two of the three characteristic tumors (parathyroids, pituitary, or pancreatic islets) is considered pathognomonic for MEN1 (23). The current clinical practice guidelines recommend three criteria for MEN1 diagnosis (22): 1) the presence of one of the MEN1-associated tumors in a first-degree relative of a patient with MEN1 syndrome; 2) the identification of a germline MEN1 mutation in a subject, who could be asymptomatic and has not yet developed radiological or biochemical signs of tumor onset; 3) the presence of two or more primary MEN1-associated endocrine tumors (pituitary, parathyroid, or pancreatic islets).

However, genetic testing in MEN1 patients meeting the clinical criteria could be negative (23, 89). Some studies demonstrated that negative testing for mutations is frequent in clinical MEN1-like presentations including a combination of pituitary and parathyroid tumors (136, 137), while GEP NET appeared more frequently and earlier in MEN1-positive probands, and its development under 30 years seems to be a predictor of a positive genetic test (24).

Biochemical screening for the MEN1 tumors onset in asymptomatic members of families with MEN1 syndrome is useful, since early diagnosis and treatment help reducing morbidity and mortality from these tumors (138). Attempts to screen for MEN1 tumors in asymptomatic relatives of affected individuals largely rely on the measurement of calcium, prolactin, IGF-I, and gastrointestinal hormones (22, 139). However, the beneficial effect of routine screening and the timing for genetic testing in pre-symptomatic individuals remain questionable (140) and psychologically stressful (141).

Medical imaging plays a key role in detection, staging, presurgical planning, and postsurgical surveillance (142). As summarized in **Table 3**, different imaging techniques are available to detect MEN1-associated tumors based on clinical practice guidelines (22). Imaging is particularly important for patients harboring a clinical diagnosis of MEN1, but showing no mutation, so as to aid distinguishing sporadic coincidental cases from true MEN1 (143). In this context, clinical observations showed that mutation-negative patients often have a more favorable clinical course (143) and may receive a less intensive follow-up to reduce radiation exposure, healthcare costs and anxiety (23). With regard to MEN1-PHPT, existing a genetic underlying predisposition to multiglandular disease, parathyroid imaging may probably result more useful to localize recurrent and/or persistent parathyroid disease after a sub-total/total parathyroidectomy (90).

**Table 3:** Imaging techniques used to detect the main MEN1-associated tumors.

Site	Tumor	Imaging
Pituitary	Pituitary tumors	MRI
Parathyroid glands	Parathyroid tumors	Neck US 99mTc-MIBI scintigraphy
		C-11 Met-PET/CT
Chest	Bronchial and thymic carcinoid	MR ICT
		Octreotide scintigraphy
		Ga-68-DOTATOC-PET/CT
		18F-FDG PET/CT
GI tract	Gastrinoma	MRI
		CT
		EUS
		SRS
		Selective abdominal angiography
		Ga-68-DOTATOC-PET/CT
	Insulinoma	MRI
		CT
		US/EUS
		SRS
		Celiac axis angiography
		GLP-1 PET/TC
	Other pNET	MRI
		CT
		EUS
		SRS
		Ga-68-DOTATOC-PET/CT
		18F-FDG PET/CT
	Non-functioning pNET	MRI
		CT
		EUS
Adrenal	Adrenal tumors	MRI or CT

CT, computed tomography; EUS, endoscopic ultrasonography; FDG, fluorodeoxyglucose; MIBI, methoxyisobutyl isonitrile; MRI, magnetic resonance imaging; PET, positron emission tomography; pNET, pancreatic neuroendocrine tumor; SRS, somatostatin receptor scintigraphy; ZES, Zollinger-Ellison syndrome; US, ultrasonography.

Surgical management remains the cornerstone of MEN1 treatment, with medical therapy being used to control hormone hypersecretion and disease symptoms depending on the tumor extension and histotype, although the antiproliferative effect of somatostatin analogs and everolimus have been repetitively shown in sporadic tumors (139, 144). In the case of multiple MEN1-associated tumors, surgical success is less frequent (22).

In MEN1-PHPT, surgery by means of subtotal or total parathyroidectomy is the treatment of choice, the latter followed by intramuscular reimplantation (brachioradialis muscle of the non-dominant forearm or sternocleidomastoid muscle) of parathyroid tissue fragments generally from the gland which shows the smallest dimensions at the neck surgery (22, 90). The optimal timing of surgery is debated and should be evaluated individually. Early surgery could be difficult because glands are minimally enlarged, which might predispose the patient to recurrence and reoperation; at the same time, longstanding hyperparathyroidism predisposes patient to more severe bone disease (145). Clinical practice guidelines recommend open bilateral neck exploration over the minimally invasive parathyroidectomy, because all the parathyroids gland are usually affected in MEN1 patients (22). If surgery is not possible because of patient’s refusal, inoperability, or negative imaging, the calcimimetic agent can be used, even if only small series showed that cinacalcet is effective in reducing serum calcium levels in MEN1 patients (43, 146, 147).

Treatments of MEN1 associated pituitary, adrenals, thymic, and bronchopulmonary tumors are similar to that for non-MEN1 tumors. However, it is important to remember that MEN1 thymic carcinoids, with a near-total prevalence in MEN1 smoker males, are associated with a very high lethality and, therefore, prophylactic thymectomy should be considered at the moment of neck surgery for MEN1-PHPT in male patients (148).

The primary treatment for GH-, ACTH-, TSH-secreting and symptomatic non-functioning pituitary tumors should involve selective transsphenoidal surgical resection if clinically feasible, with the curative intent of eradicating the tumor, or debulking the tumor mass if compressive symptoms occur (149). Treatment outcomes of these tumors in MEN1 syndrome are less successful than sporadic tumors (60). In the case of 1) patient’s inoperability, 2) post-surgical tumor residual or 3) tumor relapse, 4) unfeasible reoperation, and 5) before contemplating the use of pituitary radiotherapy, clinicians generally employ medical or third-line therapies depending on tumor type, clinical burden, individual responsiveness, local therapy availabilities, and center experience. First- or second-generation somatostatin analogs (SSAs), GH receptor antagonist and dopamine agonists are used in case of GH-secreting adenomas; while second-generation SSA, adrenolytic medications, glucocorticoid receptor-antagonist, or adrenalectomy are used for persistent Cushing’s disease. In case of TSH-omas, first-generation SSAs, dopamine agonists, or anti-thyroid medications can be used. Conversely, prolactinomas without neurological involvement are primarily treated with long-acting dopamine agonists. In the case of hypopituitarism, replacement therapy should be initiated as per individual needs.

Treatment of adrenal tumors consists of surgery for functioning tumors and non-functioning tumors with atypical characteristics, tumor size >4 cm, or significant tumor growth over a 6-months period (22, 110). In case of thymic and bronchopulmonary carcinoids, surgery is the treatment of choice. Where disease is advanced, additional therapies such as radiotherapy and chemotherapy or adrenolytic drugs should be considered (22, 110).

Surgery represents the treatment of choice also in case of functional GEP NETs. However, treatment outcomes of these tumors in MEN1 syndrome are less successful than sporadic tumors for different reasons (150):

1) MEN1 subjects often develop multiple gastrinomas, thus reducing the probability of surgical cure rates compared to similar sporadic solitary tumors. In fact, only 15% of MEN1 patients are free of disease immediately after surgery as compared to approximately 45% of non-MEN1 patients (22, 151, 152).
2) Occult metastatic disease is more frequent in MEN1 patients with NETs than in patients with sporadic endocrine tumors. For instance, a metastatic disease is present in up to 50% of subjects with MEN1-related insulinomas, whereas less than 10% of sporadic insulinomas are malignant (57).
3) MEN1-associated tumors are often larger, more aggressive, and resistant to treatment than sporadic ones. In particular, about 85% of pituitary tumors in MEN1 patients are macroadenomas, as opposed to 64% in non-MEN1 subjects, they more frequently infiltrate surrounding tissues, and show persistent hormone hypersecretion after treatment in more than 45% of cases (60, 153).

The average life expectancy in MEN1 patients with GEP NETs is reported to be shorter than in MEN1 patients without (59). However, tumor size <20 mm shows a poor tendency to grow and/or metastasize over a long monitoring period (154), irrespective of the underlying MEN1 genotype (155). In line with this evidence, other studies investigated the role of surgery *vs* surveillance on survival and liver metastatization in nonfunctioning pancreatic NETs (pNETs) (156–158) after stratification by size (≤20 *vs.* >20 mm) as well as proliferation indices, i.e. mitotic count and Ki67 (159). These authors demonstrated that MEN1 patients with small nonfunctioning pNETs (≤20 mm) can be managed by watchful waiting, hereby avoiding major surgery without loss of oncological safety (156–158).

With regards to medical therapy, a number of observational, longitudinal, and randomized placebo-controlled studies have been conducted in sporadic NETs using somatostatin analogs, peptide receptor radionuclide therapy (PRRT), (mTOR) signaling inhibitors, and receptor tyrosine kinase (RTK) inhibitors, all collectively showing a statistically significant effect on disease progression (160). Particularly PNETs are difficult to treat medically in MEN1 due to differences in growth potential, concomitant development of other tumors, and relative insensitivity to treatment, such that medical treatments for MEN1-related tumors have not been properly evaluated, but rather have been employed based on recognized effects in patients without MEN1 (161).

## Genotype-Phenotype Correlations in MEN1

A *MEN1* gene mutation can be detrimental to gene function or result in a protein product retaining residual functions. An aberrant menin protein becomes impaired in its functions through pathological interaction with transcription factors such as Smad3, JunD, and NFκB as well as nuclear receptors, or with proteins implicated in the apoptotic cascade such as caspase-3, p53, or p21 (162). Lips et al. hypothesized that MEN1 germline mutations can selectively affect menin binding to its targets and lead to distinctly aggressive clinical phenotypes (162).

Moreover, it is known that a clustering of mutations is observed in some regions of exon-2 and exon-10 which have been attributed to the nature of the underlying repetitive nucleotides prone to DNA polymerase errors (30, 56). Also, another observation of clustering of mutations at specific nucleotides in apparently unrelated families has been attributed to founder effects (29, 163–168). However, highlighting rare tumors among family members that are not seen among other individuals with the same mutation may suggest other mechanisms besides the mutation that can account for these phenotypes such as epigenetics, changes in other parts of the genome, environmental influences, immunogenicity, etc. Specifically, it is known that dysregulation of some miRNAs could account for parathyroid tumorigenesis (169–171), and this could also happen in MEN1 GEP/NETs carcinogenesis (172). Finally, epigenetic alterations could be hardly involved also in MEN1 GEP-NET tumorigenesis (173).

Although an effort has been made to characterize the potential genotype-phenotype correlation in MEN1, this link remains debated to date and no definitive evidence has been recognized (15, 25, 174). However, some authors described a heavier or lighter clinical burden in association with some specific mutations, and they will be subsequently described.

*MEN1* missense mutations have been described in association with familiar isolated hyperparathyroidism (FIHP), an autosomal dominant disease that potentially represents an early stage or a milder presentation of MEN1 attributable to an allelic variant of the *MEN1* gene (175–180). Peculiarly, most of *MEN1* gene germline mutations identified in FIHP are seemingly in-frame deletions or mild missense mutations (180).

On the other hand, studies performed in four kindreds from Newfoundland demonstrated that a single nonsense mutation in the *MEN1* gene (R460X) was predominantly associated with prolactinomas, carcinoids and parathyroid tumors (167, 181–183), though the same mutation has been described in other MEN1 cases with milder MEN1 clinical features (26, 30, 137, 164, 184–188). Therefore, there is currently no evidence of a genotype-phenotype correlation for this mutation.

In 2011, Raef and colleagues described a MEN1 family showing an aggressive tumor behavior associated with a monoallelic 5 kb deletion of genomic DNA, involving the *MEN1* promoter and exons 1 and 2 (189). LOH analysis identified a somatic deletion within the *MEN1* locus 11q13 and the 11p15 imprinting control region (ICR) of the maternal chromosome 11. Following methylation analysis of ICR, ICR1 hypermethylation and ICR2 hypomethylation were demonstrated in tumor specimens. These genetic alterations were found in association with the development of multiple malignant pNETs. Likewise, Ishida et al. described the case of a MEN1 patient with a relapsing macroprolactinoma co-stained for FSH showing histological features of malignancy and associated with a metastasizing non-functioning pNET. This phenotype was associated with menin and p27Kip1 down-regulation (190).

In 2014, Bartsch et al. retrospectively analyzed a cohort of 71 genetically confirmed MEN1 patients with the aim of evaluating the relationship between *MEN1* mutations in different interacting domains of menin and the pNETs phenotype. The authors demonstrated that patients with *MEN1* mutations leading to loss of interaction with checkpoint kinase 1 (CHES1-LOI) displayed a higher risk of malignant pNETs with an aggressive course of the disease and disease-related death (191). Longuini et al. analyzed a cohort of one hundred Brazilian MEN1 germline mutant carriers, genotyping them for the coding *p27 c.326T>G (V109G)* variant. They suggest that the p27 tumor suppressor gene could represent a disease modifier gene in MEN1 syndrome cases associated with *MEN1* germline mutations (34). Subsequently, Circelli and colleagues confirmed, in a smaller sample of 55 Italian MEN1 patients, the possible impact of this *CDKN1B* polymorphism on the clinical course of the disease, since the MEN1-related aggressive tumors, or other malignancies, were more frequent in those patients with the *CDKN1B V109G* genetic variant (35). However, currently it is still not possible to clearly state whether this p27 variant behaves as a real modifying gene or not.

In 2016, Skalniak and collegues described a three generations family with MEN1 caused by a previously undescribed in-frame deletion *c.1231_1233delGCC* (Ala411del) in the *MEN1* gene. Even if the family members differed for their phenotypic features, all NETs showed an aggressive behavior and a high mortality rate (192). Lastly, a clinical survey by Palermo et al. found a strong genotype-phenotype correlation with aggressive MEN1-related GEP NETs. In particular, the authors described three MEN1 patients carrying a novel heterozygous germline mutation in exon 10 of the *MEN1* gene, *c.1561_1571 delACTGTCGCTGG* corresponding to T521 frame-shit effect, which was associated with malignant GEP NETs lesions and a higher rate of malignancy (193). In this regard, it is correct to point out that patients with symptomatic MEN1 gastrinomas, a long-time treatment by H2-blockers or PPIs, may stimulate gastric neuroendocrine cells proliferation contributing to the clinical outcome and severity of GEP-NETs (194).

At odds with previous studies, a retrospective-prospective study on a large Italian cohort by Marini et al. analyzed *MEN1* mutation sites and features in relation to the affected menin functional domains and clinical presentations. The authors observed a wide variability in the age of disease onset and clinical severity even in the presence of the same mutation, implying a lack of direct genotype-phenotype correlations. The authors speculated that other genetic or epigenetic factors may intervene in individual MEN1 tumorigenesis (20). Nevertheless, in this large cohort a stronger association was documented between aggressive phenotypes and non-sense or frameshift mutations, than with missense mutations.

It is worth to point out that the described correlations were generally not tested in family members with the identical genotype to determine whether all of the family members had the aggressive phenotypes. Therefore, it is difficult to state whether there is a genotype-phenotype correlation between aggressive MEN1 phenotype with any type of mutation.

## A Novel MEN1 Gene Mutation Related to Aggressive Phenotype

In the context of the potential genotype-phenotype correlation, we report on the case of a 69-year-old woman with a previously unrecognized long history of MEN1, who was referred to our Unit in 2017 for complicated obesity. As common practice, patient signed an informed consent to collect clinical, biochemical, and genetic data. The investigation was approved by the local ethics committee, functioning according to the fourth edition of the Guidelines on the Practice of Ethics Committees in Medical Research With Human Participants. Unfortunately, because of the suffering for her long clinical history, not adequately managed from the beginning, and for the diagnosis that came only on the occasion of her hospitalization at our hospital unit, understandably exhausted, she did not give consent to carry out molecular studies on any of her surgically removed tissues. Her MEN1-related clinical record was noticeable for 1) a pituitary macroprolactinoma cured after trans-sphenoidal adenomectomy and external conventional radiotherapy in 1980, 2) PHPT surgically treated by excision of a single upper left parathyroid adenoma in 1981 at the age of 35 years, 3) two duodenal gastrinomas diagnosed in 2005 and left untreated according to the patient’s choice. While the diagnosis of pituitary and parathyroid disease was nearly synchronous, her osteoporotic bone involvement was severe due to dual dorsal vertebral fractures causing an early exaggerated thoracic kyphosis and compromising her biomechanical performance. Reportedly, no genetic analysis had been performed on the patient or her relatives at that stage. Upon admission to our unit, the clinical and imaging work-up showed: a normal (unstimulated) pituitary function; hypercalcemia with 3.5× elevation of PTH levels due to an apparently single left parathyroid adenoma identified at ultrasound and MIBG-scintigraphy; hypergastrinemia and high chromogranin A levels associated with endoscopic, scintigraphic (octroscan) and MRI evidence of three separate lesions located in the anterior gastric wall, in the duodenal loop, and in the pancreas head. A CT scan excluded systemic metastases. Because of associated ZES, she started proton pump-inhibitors. For her osteoporosis, she started antiresorptive treatment with oral alendronate as she refused i.v. administration with amino-bisphosphonates available by this route. After surgical and anesthesiological consultation, she underwent total parathyroidectomy and auto-transplantation of three parathyroid fragments within the brachioradialis muscle. Abdominal surgery was contraindicated for her poor clinical conditions, and she was started on octreotide LAR (30 mg/28d), leading to clinical control and near-normalization of gastrin and CgA levels. She was subsequently followed up at 3 months intervals. Due to new onset metrorrhagia, the patient was re-admitted to our unit in 2018 to undergo a diagnostic workup. Transvaginal ultrasound and pelvic MRI showed endometrial thickening, and hysteroscopic endomyometrial biopsy revealed a high-grade neuroendocrine endometrial carcinoma (G3) staining negative for gastrin and showing immunopositivity for p16, p53, CKpan, chromogranin A, vimentin, estrogen, and progesterone. Ki-67 was 50%. The patient’s conditions contraindicated gynecological surgery, and she refused palliative antineoplastic treatment.

**Figure 1:**
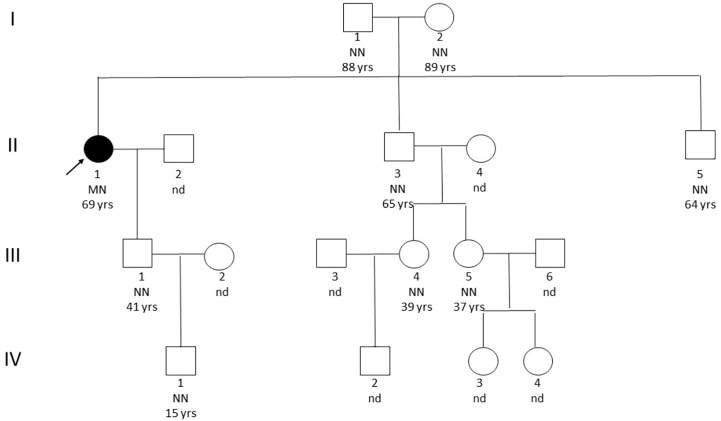
Pedigree. The black arrow indicates the affected patient. NN, homozygote without mutation; MN, heterozygous with mutation; nd, not screened.

Genetic analysis by sequencing of the coding sequence of the *MEN1* gene (NM_130799.2) and *CDKN1B* (NM_004064.3) resulted in the identification of the missense variant *c.836C>A* in exon 6 resulting in the amino acid change p.A279D in heterozygous state. This variant was not found in genomic variation databases (1000Genomes, ExAC, ESP, dbSNP, Alamut, HGMD Professional) and represented a non-conservative change leading to the switch from a hydrophobic to a basic amino acid (BLOSUM62=-2; Grantham Distance =126), located in a very conserved position in the protein domain involved in the interaction of menin protein with FANCD2. Previous in silico prediction tools of variation effects, such as SIFT, Mutation Tester, Poly Phen, Provean, and A-GVGD, suggested a probable pathogenic effect of this substitution on the aberrant patient’s phenotype. Deletions in *MEN1* gene were excluded through MLPA analysis (MRC Holland, P017-D1 probemix). Potential overlap with other published gene mutations sharing the same amino acid sequence or functional domain effects was also excluded. We subsequently evaluated the co-segregation of the afore-mentioned *MEN1* variant in the proband family (**Figure 1**). A total of 16 relatives, including her old parents, were screened but none was found to carry the index mutation or any other variant, likewise none was presenting clinical manifestations suggestive of MEN1. Further, the possible contribution to the proband phenotype of *BRCA1/2* mutations was excluded through NGS (Illumina, NY, USA) and MLPA analysis (MRC Holland, P002-D1 BRCA1 and P090-B1 BRCA2 probe mix).

## Discussion

Clinical guidelines focus on early detection of MEN1-related tumors, namely parathyroid adenomas, GEP NETs, and pituitary adenomas (22, 139, 144). An aggressive or aberrant behavior of endocrine tumors has been occasionally described in patients with MEN1 syndrome, raising the following questions: do some MEN1 patients with specific mutations carry an increased risk of aberrant clinical progression requiring in-depth diagnostic and therapeutic assessment? Also, are tumor-related manifestations or the disease course dependent on the type of mutation? Earlier studies failed to show strong genotype-phenotype associations, while more novel studies seem to dispute this viewpoint. It is suggestive to speculate that the type of *MEN1* gene mutation could influence the clinical manifestations of MEN1. In MEN1 syndrome exhibiting a non-aggressive phenotype, frameshift or nonsense leading to a truncated and consequently inactivated protein have been identified in most cases (120). Likewise, FIHP is characterized by the onset of primary hyperparathyroidism alone and is related to specific mutations of *MEN1* gene that often include missense mutations and only occasionally nonsense or frameshift mutations (22, 89). Conversely, some specific mutations seem to be associated with a less favorable prognosis. For example, subjects with *MEN1* mutations leading to a loss of interaction with the checkpoint kinase 1-interacting domain have a higher risk of malignant pNETs with aggressive phenotype and higher prevalence of disease-related death (192). Peculiarly, patients with mutations that affect the JunD-interacting domain have a higher risk of death for a typical MEN1 tumor, requiring a more aggressive therapeutic approach (195). In keeping with these indications, the novel missense variant herein reported *c.836C>A* resulting in the amino acid change p.A279D in heterozygous state, leads to a change from alanine to aspartic acid with potential aggressive behavior. At odds with studies minimizing the clinical impact of missense mutations compared to frameshift or non-sense mutations (20, 24), the missense variant described in our index case stands out for its aberrant and aggressive clinical manifestations developing long after the first clinical manifestation of MEN1.

## Conclusions

Current clinical practice guidelines for MEN1 recommend a screening program for MEN1 patients and their families with the aim of reducing morbidity and mortality and achieving an early detection of MEN1-related tumors (22, 139). Although genotype-phenotype correlations are difficult to demonstrate, our index case and other reports suggest that patients with suggestive genotype-phenotype correlations should undergo a closer follow-up and surveillance with an interdisciplinary approach. In fact, our team was involved and intervened only at a relatively advanced stage in the clinical history of this case, when the patient was understandably exhausted and did not give consent to carry out molecular studies on her surgically removed tissues. Consequently, we lack molecular data on menin expression and function, LOH studies at the tumor tissue level, as also on possible involvement of specific miRNAs. This aspect further confirms the importance that subjects affected by rare and complex pathologies such this are taken in charge from the beginning by expert multidisciplinary teams, also capable of managing the psychological aspects and implications linked to genetic and repeatedly clinical management complexity.

## Author Contributions

Conceptualization and methodology, CM, MM, PM. Original draft preparation, CM, MM, MC, AF, SM. Review and editing, CM, LP, AF, PM. Supervision, GA, MS, AF. All authors contributed to the article and approved the submitted version.

## Conflict of Interest

The authors declare that the research was conducted in the absence of any commercial or financial relationships that could be construed as a potential conflict of interest.
